# Qualitative Analysis of Chemical Composition Changes Before and After *Lactobacillus* Fermented *Ziziphi Spinosae* Semen by LC‐MS and Prediction of Fermentation Mechanism

**DOI:** 10.1002/cbdv.71686

**Published:** 2026-09-23

**Authors:** Jiafu Wan, Yunda Wang, Xiaoxue Yang, Qingmei Guo

**Affiliations:** ^1^ School of Pharmacy Shandong University of Traditional Chinese Medicine Jinan P. R. China; ^2^ Key Laboratory of Traditional Chinese Medicine Classical Theory Ministry of Education Shandong University of Traditional Chinese Medicine P. R. China; ^3^ Shandong Key Laboratory of Digital Traditional Chinese Medicine Shandong University of Traditional Chinese Medicine P. R. China

**Keywords:** fermentation, HPLC, *Lactobacillus*, UPLC‐Q‐Exactive Orbitrap‐MS, *Ziziphi Spinosae* Semen

## Abstract

This study aimed to screen dominant *Lactobacillus* strains for fermenting *Ziziphi Spinosae* Semen (ZSS), analyze chemical composition changes before and after fermentation, and speculate on the fermentation mechanism. Liquid fermentation of ZSS was conducted using *Lactobacillus plantarum*, *Lactobacillus paracasei*, *Lactobacillus acidophilus*, and *Lactobacillus rhamnosus*. HPLC was employed to determine the content of spinosin, 6′′′‐feruloylspinosin, jujuboside A, and jujuboside B. Total phenolic content (TPC), total flavonoid content (TFC), and antioxidant capacity were measured using UV spectrophotometry. Principal component analysis (PCA) was employed to identify the optimal fermentation strain, and UPLC‐Q‐Exactive Orbitrap‐MS technology was used for qualitative analysis of chemical components before and after fermentation. Results indicated *L. plantarum* as the optimal fermentation strain. UPLC‐Q‐Exactive Orbitrap‐MS analysis revealed 41 chemical components in the unfermented sample, reduced to 30 post‐fermentation. *L. plantarum* was hypothesized to catalyze enzymatic hydrolysis breaking C─O bonds, N─C bonds, acyl groups, and glucosyl groups, facilitating interconversion among compounds.

## Graphical abstract

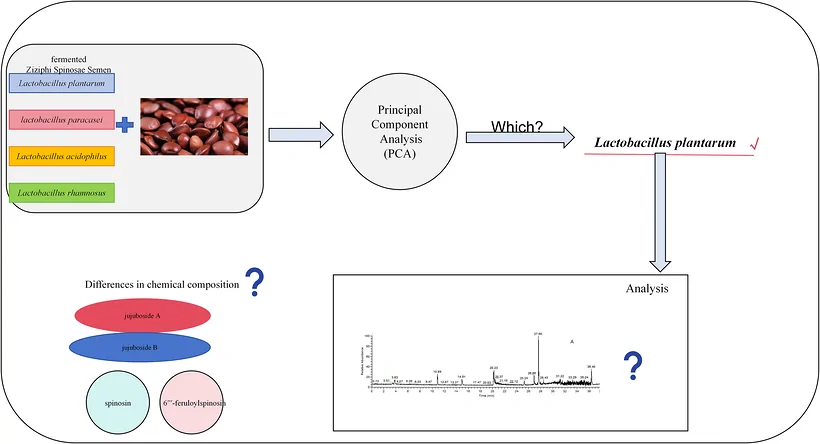

## 1 Introduction

In China, *Ziziphi Spinosae* Semen (ZSS) has served as the primary herbal remedy for insomnia and anxiety in clinical settings for over two millennia [1, 2, 3]. ZSS is the dried mature seed of *Ziziphus jujuba* Mill var. *spinosa* (Bunge) Hu ex H.F.Chow., a plant belonging to the Rhamnaceae family and the genus *Ziziphus*. Modern research indicates that ZSS possess a complex chemical composition, including saponins, triterpenoids, flavonoids, alkaloids, polysaccharides, and proteins [4, 5, 6]. The Pharmacopoeia of the People's Republic of China uses spinosin and jujuboside A in ZSS as quality control standards. Jujuboside A in ZSS can inhibit inflammatory responses and oxidative stress in nonalcoholic fatty liver disease, potentially serving as a therapeutic agent for this condition [7]. Spinosin can alleviate Alzheimer's disease‐associated hippocampal synaptic deficits through BDNF/TrkB signaling pathways. It also mitigates liver fibrosis by inhibiting activated hepatic stellate cells via targeting the Nur77/ASK1/p38 MAPK signaling pathway. Additionally, ZSS extract improves rat insomnia by regulating metabolomics and gut microbiota composition [8]. ZSS possess multiple pharmacological effects, including sedation and sleep promotion, anti‐anxiety, anti‐depression, and antioxidant properties [9]. They hold promising prospects for the development of novel drugs, health products, and functional foods.

Fermentation is a biochemical process that utilizes the metabolic functions (including microorganisms and plant cells) of organisms to decompose organic matter. As a traditional method, fermentation has been applied in both food and pharmaceutical aspects of human life. In ancient China, fermentation techniques were applied to brew alcohol, and later, yeast was incorporated into herbal preparations. Fermentation gradually evolved into a crucial technology in traditional Chinese medicine, giving rise to various fermented medicinal substances such as Jiao San Xian, Shen Qu, and Ban Xia Qu [10]. Shen Qu possesses biological effects that strengthen the spleen and stomach while promoting digestion, making it effective for treating gastrointestinal disorders [11]. Many active components in traditional Chinese medicine belong to secondary metabolites, which are present in low concentrations within the herbs. Fermentation technology can increase the content of these natural medicinal compounds to meet therapeutic demands. During the fermentation of traditional Chinese medicines, microorganisms generate or enhance secondary metabolites through chemical reactions such as esterification, acylation, and glycosylation. Simultaneously, they break down plant cell walls, thereby increasing drug solubility. *Streptococcus lactis* can effectively degrade cellulose. *Streptococcus lactis* fermentation of Astragalus membranaceus increased the total flavonoid and total saponin content in its roots, stems, and leaves [12]. Additionally, natural products are not readily absorbed and utilized by the human body. However, through fermentation technology, they can be converted into components that are easily absorbed and utilized [11], thereby enhancing the bioavailability of drugs and improving therapeutic efficacy [13]. Certain natural compounds in traditional Chinese medicine can promote the proliferation of beneficial bacteria in the human body. *Red Ginseng* and *Semen Coicis* have been shown to stimulate the growth of *Bifidobacterium* and *Lactobacillus* in vitro, thereby alleviating symptoms of ulcerative colitis [14]. Therefore, the synergistic effect of traditional Chinese medicine and probiotics can enhance the efficacy of traditional Chinese medicine.

In recent years, lactic acid bacteria have been widely used in the fermentation and preparation of food and pharmaceutical products. They enhance the properties of natural products, exhibit antioxidant and antibacterial activities, and improve the appearance and quality of food products [15]. *L. rhamnosus* (F‐B4‐1) and *Bacillus subtillis Natto* (F‐A7‐1) fermentation of Salvia miltiorrhiza effectively alleviates dextran sulfate sodium‐induced ulcerative colitis in mice [16]. For instance, salicin possesses an ortho‐phenolic hydroxyl group, resulting in poor lipid solubility. Fermentation converts water‐soluble components in *Salvia miltiorrhiza* into lipid‐soluble forms, enhancing the absorption of active compounds in the intestinal tract. Fermenting apple juice using a blend of *L. acidophilus*, *L. plantarum*, and *Lactobacillus fermentum* enhances the juice's palatability and intensifies its aroma [17]. *Lactobacillus paracasei* and *L. plantarum*, when fermenting prickly pear juice separately, both significantly enhance the juice's antioxidant capacity and α‐glucosidase inhibitory activity. Each lactic acid bacterium exhibits distinct metabolic characteristics: *L. plantarum* exerts a more pronounced effect on organic acids and phenolic compounds, while *L. paracasei* demonstrated superiority over *L. plantarum* in upregulating amino acid and nucleotide levels along with their derivatives [18]. The combined application of protein hydrolysis and *L. plantarum* fermentation significantly enhances the antioxidant activity of quinoa beverages. During fermentation, *L. plantarum* produces lactic acid and various enzymes that degrade plant cell wall structures, thereby increasing the extraction of bioactive compounds such as flavonoids and phenolic acids [19]. Cinnamon fermented with *L. plantarum* exhibits enhanced antioxidant activity, increased anticancer activity, and higher levels of total phenols and total flavonoids, without producing toxic substances [20].

This study employed various *Lactobacillus* strains to ferment ZSS. The major bioactive constituents responsible for the sedative and anxiolytic effects of ZSS—jujuboside A, jujuboside B, spinosin, and 6′′′‐feruloylspinosin—together with total flavonoid content (TFC), total phenolic content (TPC), and antioxidant capacity, were used as screening criteria for the selection of optimal fermentation strains. Principal component analysis (PCA) was applied to integrate these multiple indicators into a comprehensive evaluation, providing a more holistic and objective strategy than single‐index comparisons. UPLC‐Q‐Exactive Orbitrap‐MS was further employed to qualitatively analyze the differences in chemical composition before and after fermentation, and the enzymatic mechanism underlying *L. plantarum* fermentation of ZSS was proposed.

## 2 Materials and Methods

### 2.1 Herbs and Strains

ZSS was purchased from Hebei a herbal market in Anguo, Hebei Province, china, and originated from Xingtai, Hebei Province (harvested in October 2020). The botanical identity was authenticated by Prof. Zhou Fengqin (Shandong University of Traditional Chinese Medicine) as the dried ripe seeds of *Ziziphus jujuba* Mill. var. *spinosa* (Bunge) Hu ex H. F. Chou (Rhamnaceae).

*L. plantarum* (20191112) was purchased from Shanghai Conservation Biotechnology Center. *L. paracasei* (20042723) was purchased from Beijing Beinanchuanglian Biotechnology Research Institute. *L. acidophilus* and *L. rhamnosus* were purchased from Food Biotechnology Laboratory of Jiangnan University.

### 2.2 Reagents

MRS broth, MRS agar, and potato‐dextrose agar (PDA) were purchased from Beijing Landbridge Technology Co., Ltd. YPD/YEPD medium powder was purchased from Shanghai Yuanye Biotechnology Co., Ltd. The reference standards of jujuboside A, jujuboside B, spinosin, 6′′′‐feruloylspinosin, gallic acid, rutin, Folin‐ciocalteu reagent, and ascorbic acid were also sourced from Shanghai Yuanye Biotechnology Co., Ltd. Formic acid, acetonitrile (chromatography grade) were purchased from Thermo Fisher Scientific. Watson's distilled water was procured from Watson's Co., Ltd.

### 2.3 Strain Activation

MRS medium was prepared by dissolving 5.52 g of MRS broth with in 100 mL of distilled water. Single *Lactobacillus* strains were inoculated into MRS medium and incubated at 37°C for 12 h. For composite cultures, were prepared at the following ratios: *L. acidophilus*: *L. rhamnosus* (1:1), *L. plantarum*: *L. paracasei* (1:1), *L. plantarum*: *L. acidophilus*: *L. rhamnosus*: *L. paracasei* (1:1:1:1), inoculated into MRS medium, and incubated under the same conditions.

### 2.4 Liquid Fermentation of ZSS

ZSS powder (10 g) that had been passed through a 60‐mesh sieve was transferred into a conical flask. The fermentation medium was prepared according to the method described in [21] with a solid‐to‐liquid ratio of 1:10, pH 4.2–4.5, and the carbon source was adjusted to 17% by adding isomaltulose. The medium was sterilized by autoclaving at 121°C for 30 min and allowed to cool to room temperature. The sterilized medium was then inoculated with 2.5%(v/v) of either single strains of *L. plantarum*, *L. acidophilus*, *L. rhamnosus*, *L. paracasei*, or composite strains mixed at the following ratios: *L. plantarum*: *L. paracasei* = 1:1, *L. acidophilus*: *L. rhamnosus* = 1:1, *L. plantarum*: *L. acidophilus*: *L. rhamnosus*: *L. paracasei* = 1:1:1:1. The flasks were incubated anaerobically at 37°C for 72 h. After fermentation, the cultures were pasteurized at 90°C for 30 min and then centrifuged at 4000 r/min for 20 min. The resultingsupernatant was collected. The sample designations are listed in Table 1.

**TABLE 1:** Lactobacillus fermentation sample numbers.

Serial number	Fermentation strains
1	*L. acidophilus*
2	*L. rhamnosus*
3	*L. plantarum*
4	*L. paracasei*
5	*L. acidophilus*: *L. rhamnosus* = 1:1
6	*L. plantarum*: *L. paracasei* = 1:1
7	*L. plantarum*: *L. acidophilus*: *L. rhamnosus*: *L. paracasei* = 1:1:1:1

### 2.5 Preparation of Unfermented Samples

ZSS powder (10 g) that had been passed through a 60‐mesh sieve was mixed with distilled water at a solid‐to‐liquid ratio of 1:10. The mixture was autoclaved at 121°C for 30 min and allowed to cool to room temperature. Without inoculation of any *Lactobacillus* strains, the flask was placed in a constant‐temperature incubator at 37°C and incubated anaerobically for 72 h under the same conditions as the fermented samples. After incubation, the mixture was pasteurized at 90°C for 30 min, sonicated for 40 min, and then centrifuged at 4000 r/min for 20 min. The supernatant was collected as the unfermented sample.

### 2.6 Determination of Spinosin, 6′′′‐Feruloylspinosin, Jujuboside A, and Jujuboside B by Hplc

#### 2.6.1 Sample Preparation

The fermented and unfermented samples were prepared according to the procedures described in Sections 2.4 and 2.5, respectively.

#### 2.6.2 Preparation of Reference Standard Solutions

Reference Methods [4, 22]. Individual stock solutions of the four reference standards were prepared by accurately weighing the appropriate amounts and dissolving them in methanol. The final concentrations were 0.462 mg/mL for jujuboside A, 0.494 mg/mL for jujuboside B, 0.426 mg/mL for spinosin, and 0.602 mg/mL for 6′′′‐feruloylspinosin.

#### 2.6.3 Chromatographic Conditions

Chromatographic separation was performed on an Agilent 5 TC‐C18 column (250 mm × 4.6 mm, 5 µm). The mobile phase consisted of acetonitrile (A) and 0.15% acetic acid in water (B). The gradient elution program was as follows: 82% B (0–10 min); 82%→70% B (10–25 min);70%→10% B (25–45 min); 10%→82% A (45–50 min). The flow rate was maintained at 1 mL/min throughout the analysis. The column temperature was set at 30°C, and the injection volume was 20 µL. Spinosin and 6′′′‐feruloylspinosin were detected by DAD at 335 nm, while jujuboside A and jujuboside B were detected by ELSD with the following parameters: drift tube temperature, 100°C; nebulizer temperature, 85°C; and nitrogen gas flow rate, 1.6 L/min. The identity of the four target compounds in the samples was confirmed by comparing their retention times and UV spectra with those of the authentic reference standards.

#### 2.6.4 Method Validation

The analytical method was validated in terms of linearity, precision, stability, repeatability, and accuracy.

Linearity: Working standard solutions were prepared by diluting the stock solutions with methanol to obtain a series of concentrations. For spinosin and 6′′′‐feruloylspinosin, the calibration curves were constructed by plotting the peak area (y) against the concentration (x). For jujuboside A and jujuboside B, the calibration curves were established using the logarithm of peak area versus the logarithm of concentration. The regression equations, linear ranges, and correlation coefficients (R^2^) are summarized in Table 2.

**TABLE 2:** Calibration curves, linear ranges, and correlation coefficients of the four analytes.

Analyte	Regression equation	R^2^	Linear range (mg/mL)
Spinosin	y = 16981x−36.62	0.9994	0.03408–0.1704
6′′′‐Feruloylspinosin	y = 14183x−20.12	0.9993	0.02408–0.1204
Jujuboside A	y = 1.4732x+3.6028	0.9991	0.01848–0.0924
Jujuboside B	y = 1.6446x+3.8925	0.9992	0.01260–0.1260

Precision: The precision was evaluated by performing six replicate injections of the same mixed reference standard solution. The relative standard deviation (RSD) of peak areas was 1.19% (< 3%), demonstrating satisfactory instrument precision.

Stability: The stability of the sample solution was assessed by analyzing the same fermented sample at 0, 6, 12, 24, 48, and 72 h. The RSD of peak areas was 1.63% (< 3%), indicating that the sample solution was stable within 72 h.

Repeatability: Six independently prepared aliquots of the fermented sample were analyzed in parallel. The RSD of peak areas was 1.60% (< 3%), confirming good repeatability of the method.

Recovery: Known amounts of the four reference standards were spiked into six aliquots of the fermented sample. The recoveries and RSD values were calculated. The results (Table 3) confirmed that the method met the requirements for the quantitative determination of the four analytes.

**TABLE 3:** Recovery of the four analytes.

Analyte	Recovery (%)	Recovery (RSD, %)
Spinosin	99.55%	1.30%
6′′′‐Feruloylspinosin	99.56%	1.52%
Jujuboside A	100.49%	1.55%
Jujuboside B	100.86%	1.43%

#### 2.6.5 Determination of Samples

**FIGURE 1:**
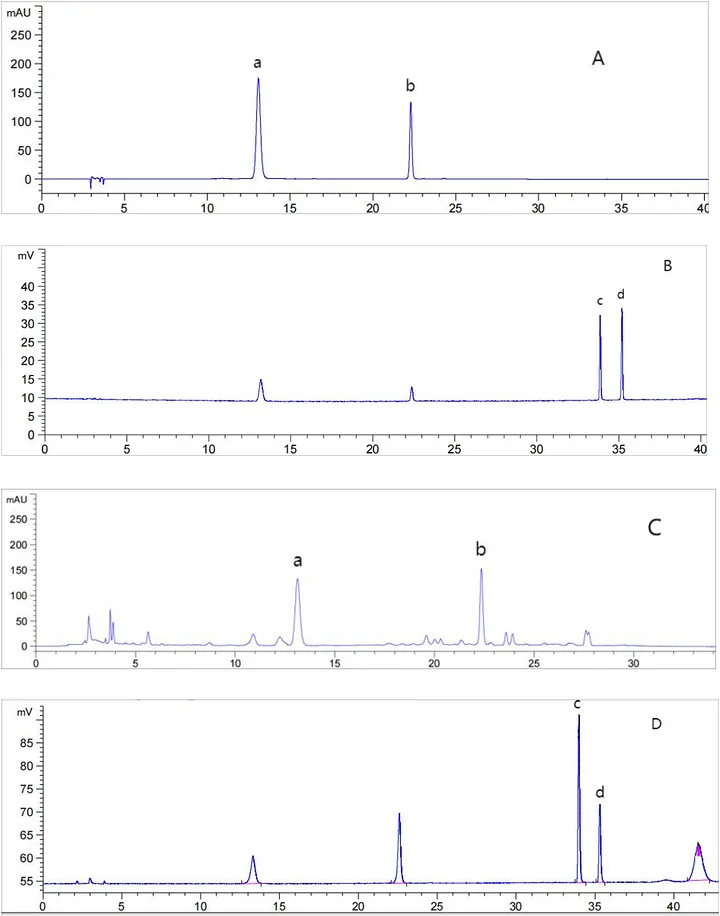
Representative chromatograms of the reference standards and samples. (a) spinosin, (b) 6′′′‐feruloylspinosin, (c) jujuboside A, (d) jujuboside B. Panels (A) and (C) were detected by DAD at 335 nm; panels (B) and (D) were detected by ELSD.

The test solution was filtered through a 0.45 µm microporous membrane, and the resulting filtrate was injected into the HPLC system. The peak areas of spinosin, 6′′′‐feruloylspinosin, jujuboside A, and jujuboside B were recorded, and their contents were calculated from the corresponding regression equations established in Section 2.6.4. The results were expressed as milligrams per gram of original herb (mg/g). Representative chromatograms of the reference standards and samples are shown in Figure 1 (see also Figure S1).

### 2.7 Total Phenolic Content (TPC) Determination

Reference Methods [23]. A 100 µL aliquot of the fermentation sample was diluted with distilled water to a final volume of 2.5 mL. Subsequently, 1 mL of Folin–Ciocalteu reagent and 1.5 mL of 15% Na_2_CO_3_ solution were added. The mixture was vortexed and incubated at room temperature in the dark for 30 min. The absorbance was measured at 760 nm. A standard curve was constructed using gallic acid standard solutions at concentrations of 2.884, 4.326, 5.768, 7.21, 8.652, and 10.094 µg/mL. The TPC was calculated from the regression equation and expressed as milligrams of gallic acid equivalents per gram of original herb (mg/g).

### 2.8 Total Flavonoid Content (TFC) Determination

Reference Method [24]. TPC was determined using a colorimetric method with rutin as the reference standard. Briefly, a suitable volume of the sample or standard solution was mixed with 0.15 mL of 5% NaNO_2_ solution. After incubation for 6 min at room temperature, 0.15 mL of 10% Al(NO_3_)_3_ solution was added and the mixture was allowed to react for another 6 min. Subsequently, 2 mL of 4% NaOH solution was added, followed by dilution to a fixed volume with 70% ethanol. The mixture was thoroughly mixed and incubated at room temperature for 15 min. The absorbance was measured at 510 nm. A calibration curve was established by plotting the absorbance values against the concentrations of rutin standard solutions. The TFC in the samples was calculated from the regression equation and expressed as milligrams of rutin equivalents per gram of original herb (mg/g).

### 2.9 Antioxidant Activity

#### 2.9.1 Measurement of DPPH Radical Scavenging Activity

Reference method [25]. The DPPH radical scavenging activity was measured according to a previously reported method with slight modifications. Briefly, 200 µL of ascorbic acid standard solution (or appropriately diluted sample) was mixed with 2 mL of freshly prepared DPPH solution (0.2 mmol/L in ethanol). The mixture was incubated in the dark at room temperature for 30 min, and the absorbance was measured at 517 nm. The DPPH radical scavenging rate was calculated using the following formula:

$$\mathrm{DPPH}\;\mathrm{scavenging}\;\mathrm{activity}\left(\%\right)=[1-(A_{i}-A_{j})/A_{c}]\times 100\%$$

where A_i_ is the absorbance of the sample mixed with DPPH ethanol solution, A_j_ is the absorbance of the sample mixed with anhydrous ethanol (sample blank), and A_c_ is the absorbance of the control (DPPH ethanol solution mixed with distilled water instead of sample).

#### 2.9.2 Determination of Hydroxyl Radical Scavenging Activity (HRSA)

Reference method [26]. The hydroxyl radical scavenging activity was determined according to a previously reported method with slight modifications. Briefly, 0.3 mL of 6 mmol/L FeSO_4_ solution was mixed thoroughly with 0.3 mL of 6 mmol/L H_2_O_2_ and incubated in the dark at room temperature for 10 min to generate hydroxyl radicals via the Fenton reaction. Subsequently, 1 mL of sample (or ascorbic acid standard) solution was added and allowed to react for 10 min. Finally, 0.3 mL of 6 mmol/L salicylic acid was added, and the mixture was incubated in the dark at 37°C for 30 min. The absorbance was measured at 510 nm, and the hydroxyl radical scavenging rate was calculated using the following formula:

$$\text{Hydroxyl radical scavenging activity}\left(\%\right)=\left[1-\frac{A_{1}-A_{2}}{A_{0}}\right]\times 100\%$$

where A_1_ is the absorbance of the sample mixed with FeSO_4_ solution, H_2_O_2_ solution, and salicylic acid; A_2_ is the absorbance of the sample mixed with FeSO_4_ solution, H_2_O_2_ solution, and distilled water (instead of salicylic acid, serving as the sample background control); and A_0_ is the absorbance of the blank control, in which distilled water replaces the sample solution, mixed with FeSO_4_, H_2_O_2_, and salicylic acid.

#### 2.9.3 Determination of Total Reducing Capacity (FRAP)

Reference method [27, 28]. The FRAP working solution was freshly prepared by mixing 20 mmol/L FeCl_3_ solution, 10 mmol/L TPTZ solution (dissolved in 40 mmol/L hydrochloric acid), and 0.3 mol/L acetate buffer (pH 3.6) in a volume ratio of 1:1:10. To construct the calibration curve, a FeSO_4_·7H_2_O stock solution (1 mg/mL) was precisely diluted with distilled water to obtain a series of working standard solutions within the concentration range of 0.05–0.4 mg/mL. A 300 µL aliquot of each standard solution was mixed thoroughly with 3 mL of the FRAP working solution. The mixture was incubated in a water bath at 37°C for 10 min, and the absorbance was measured at 593 nm. The regression equation was established as y = 1.8008x + 0.014 (r = 0.9992), indicating good linearity. For sample analysis, the fermented and unfermented samples were processed under the same conditions, and the FRAP values were calculated from the regression equation. The results were expressed as mmol Fe^2^^+^ per gram of original herb (mmol Fe^2^^+^/g).

### 2.10 Screening of Optimal Fermentation Strains

PCA was performed using OriginPro 2024 software. Nine indicators were selected as variables, including the contents of spinosin, 6′′′‐feruloylspinosin, jujuboside A, jujuboside B, TPC, TFC, DPPH, hydroxyl radical scavenging activity (HRSA), and ferric reducing antioxidant power (FRAP). The raw data were first standardized to eliminate the influence of different measurement scales. Principal components with eigenvalues greater than 1 were extracted. The first two principal components (PC1 and PC2), which together accounted for the majority of the total variance, were used for score plot and loading plot visualization. The composite score for each fermentation group was calculated from all extracted principal components weighted by their respective variance contribution rates. The optimal fermentation strain or combination was determined based on the highest composite score.

### 2.11 Pearson Correlation Analysis

Pearson correlation analysis was performed using Origin Pro 2024 to evaluate the relationships among bioactive components (jujuboside A, jujuboside B, spinosin, 6'''‐feruloylspinosin, TPC, TFC) and antioxidant activities (DPPH, HRSA, FRAP). Results were visualized as a heatmap with a color gradient from blue (negative correlation) to red (positive correlation). Statistical significance was denoted as *: p< 0.05, **: p < 0.01, and ***: p < 0.001.

### 2.12 UPLC‐Q‐Exactive Orbitrap‐MS Analysis

#### 2.12.1 Preparation of Test Solutions

The unfermented control sample was prepared as described in Section 2.5. The fermented samples were prepared using the optimal microbial strain or combination selected in Section 2.10, following the fermentation procedure detailed in Section 2.4.

#### 2.12.2 Preparation of Mixed Reference Solution for LC‐MS Analysis

Spinosin (1.41 mg), 6′′′‐feruloylspinosin (1.16 mg), jujuboside A (1.61 mg), jujuboside B (1.98 mg), and betulinic acid (1.30 mg) were precisely weighed and transferred into a 5 mL volumetric flask. The mixture was dissolved and diluted to volume with acetonitrile to obtain the mixed reference solution for subsequent Orbitrap‐MS analysis.

#### 2.12.3 Chromatographic Conditions

Chromatographic separation was performed on an ACQUITY UPLC HSS T3 column (100 mm × 2.1 mm, 1.8 µm) using a mobile phase consisting of 0.05% formic acid in acetonitrile (A) and 0.05% formic acid in water (B). The linear gradient program was set as follows: 98%→83% B (0–10 min), 83% B (10–12 min), 83%→81% B (12–14 min), 81%→67% B (14–25 min), 67%→0% B (25–30 min), and 0% B (30–35 min). The injection volume was 5 µL, the column temperature was maintained at 25°C, and the flow rate was set at 0.3 mL/min.

#### 2.12.4 UPLC‐Q‐Exactive Orbitrap‐MS Mass Spectrometry Conditions

Mass spectrometric analysis was performed on a Q‐Exactive Orbitrap mass spectrometer equipped with an electrospray ionization (ESI) source operated in both positive and negative ion modes. The optimized parameters were set as follows: sheath gas flow rate, 13.5 L/min; auxiliary gas flow rate, 3 L/min; spray voltage, 3 kV; capillary temperature, 350°C; auxiliary gas temperature, 350°C; and collision energy, 60 eV. The acquisition was performed in Full MS/dd‐MS^2^ (data‐dependent MS^2^) mode over a mass scan range of m/z 80–1200.

#### 2.12.5 Establishment of the ZSS Chemical Composition Database

A chemical composition database for ZSS was established by systematically searching and compiling information from both domestic and international databases, including CNKI, PubMed, PubChem, and Chemical Book. The collected data encompassed the English names of compounds, molecular formulas (and/or structural formulas), accurate precursor ion masses (m/z), CAS registry numbers, MS/MS fragment ions, and relevant references.

#### 2.12.6 Identification of Chemical Constituents

For compounds with available reference standards, identification was confirmed by matching the retention times, accurate precursor ion masses, and MS/MS fragment ions with those of the authentic standards.

For compounds lacking reference standards, tentative identification was performed using the in‐house ZSS chemical composition database established in Section 2.11.5. Total ion current (TIC) chromatograms acquired in both positive and negative ion modes were processed using the Xcalibur 3.0 software. The molecular formula was assigned by comparing the measured accurate mass of the precursor ion with the theoretical mass, with a mass tolerance of ≤ 10 ppm (calculated as: deviation (ppm) = (measured value − theoretical value) / theoretical value × 10^6^). Structural identification was further supported by interpretation of the MS/MS fragmentation patterns and comparison with literature data compiled in the database.

## 3 Results and Discussion

### 3.1 Changes in TPC and TFC Contents During Liquid Fermentation of Different *Lactobacillus* spp

**FIGURE 2:**
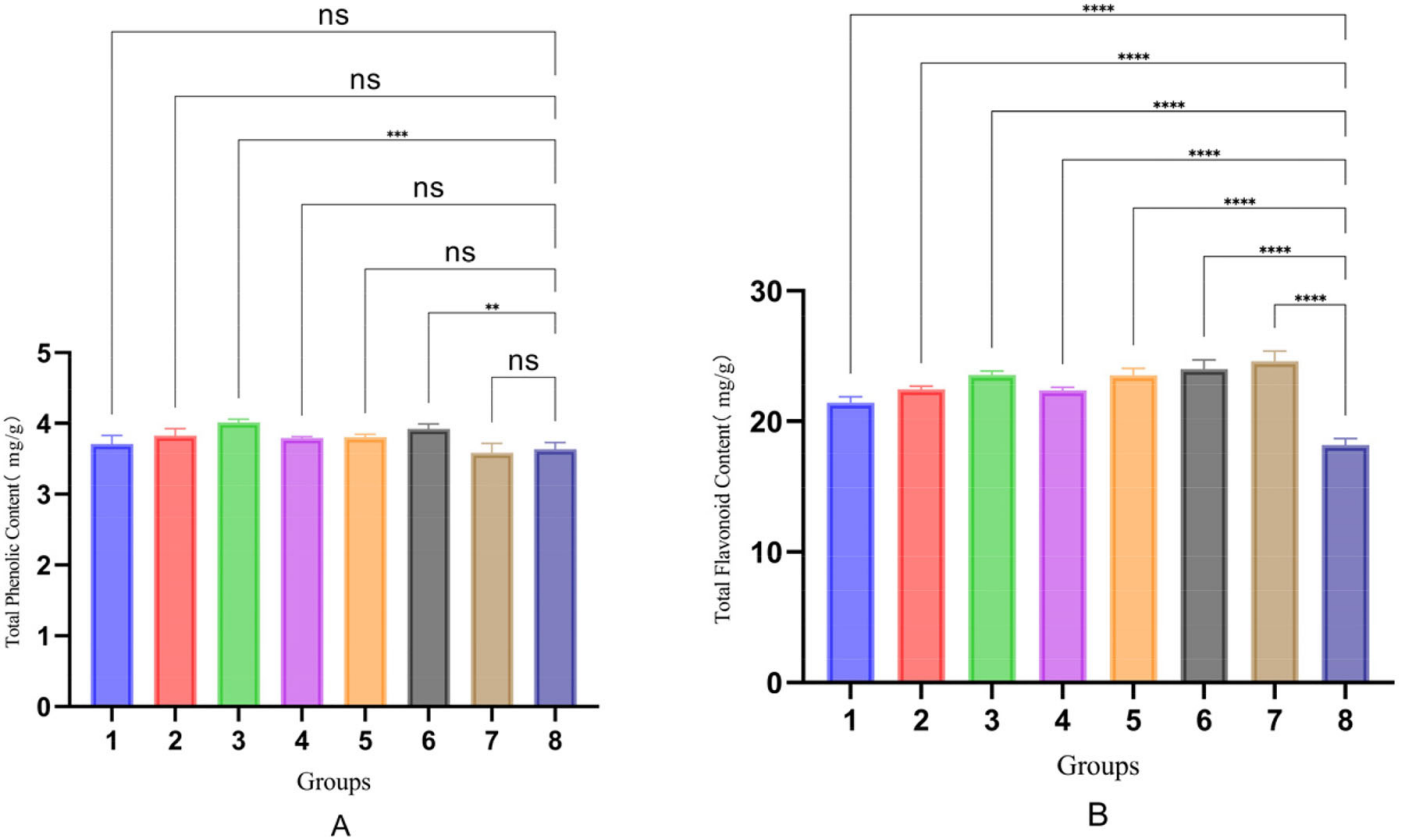
Changes of TPC and TFC contents before and after fermentation of ZSS with different strain combinations. 1: *L. acidophilus*; 2: *L. rhamnosus*; 3: *L. plantarum*; 4: *L. paracasei*; 5: *L. acidophilus*: *L. rhamnosus* = 1:1; 6: *L. plantarum*: *L. paracasei* = 1:1; 7: *L. plantarum*: *L. acidophilus*: *L. rhamnosus*: *L. paracasei* = 1:1:1:1 (compared to unfermented sample 8, *p<0.05, **p < 0.01, and ***p < 0.001).

Changes in TPC are shown in Figure 2A. Except for sample No. 7 from the composite microbial strain, which exhibited lower TPC than the unfermented sample, all other fermented samples demonstrated higher TPC than the unfermented sample. The unfermented sample contained 3.6354 ± 0.08 mg/g of TPC, while fermented samples ranged from 3.5833 ± 0.11 mg/g to 4.0760 ± 0.12 mg/g. Post‐fermentation, TPC in fermented ZSS increased by 2.09‐12.12% compared to unfermented levels, except for sample No.7, which showed a decrease relative to its unfermented counterpart. Liu et al. [29] reported a significant increase in TPC (p < 0.05) in goji berry juice after mixed fermentation with *Bacillus velezensis*, *Bacillus licheniformis*, *Lactobacillus reuteri*, *L. rhamnosus*, and *L. plantarum*. They suggested that *L. rhamnosus*, *L. plantarum*, and *Lactobacillus casei* produce acidic substances and enzymes such as glycosidases and esterases that disrupt the plant cell wall structure, thereby releasing bound phenolic compounds. In the present study, multiple *Lactobacillus* strains were used to ferment ZSS. Similarly, these microorganisms can produce glucosidase, rhamnosidase, feruloyl esterase, and other enzymes that may transform phenolic compounds within ZSS.

Changes in TFC are shown in Figure 2B. Compared to unfermented samples, TFC increased to varying degrees in samples fermented by different bacterial strains. Sample No. 7 exhibited a TFC of 24.6014 ± 0.5673 mg/g, representing a 35.19% increase (*p* < 0.05). TFC in samples fermented by different *Lactobacillus* strains increased by 17.73–35.19% compared to unfermented samples. Li et al. [30] employed lactic acid bacteria fermentation to enhance the bioactive compounds in Bian Que's Three Bean Soup. Total flavonoids increased significantly by 31.5% under lactic acid bacteria metabolism. In plants, flavonoid compounds primarily exist in bound forms. Lactic acid bacteria release more soluble bioactive compounds—such as flavonoids, isoflavones, and phenolic compounds—by hydrolyzing chemical bonds between flavonoids, phenolics, and cell wall structural components. This process enhances both the content and bioactivity of flavonoids and phenolics in Bian Que's Three Bean Soup. Enzymes produced by Lactobacillus can break down plant cell walls to release more flavonoid compounds or hydrolyze glycosides to generate numerous aglycones, thereby increasing flavonoid content.

### 3.2 Changes in Spinosin, 6′′′‐feruloylspinosin, Jujuboside A, and Jujuboside B Content

**FIGURE 3:**
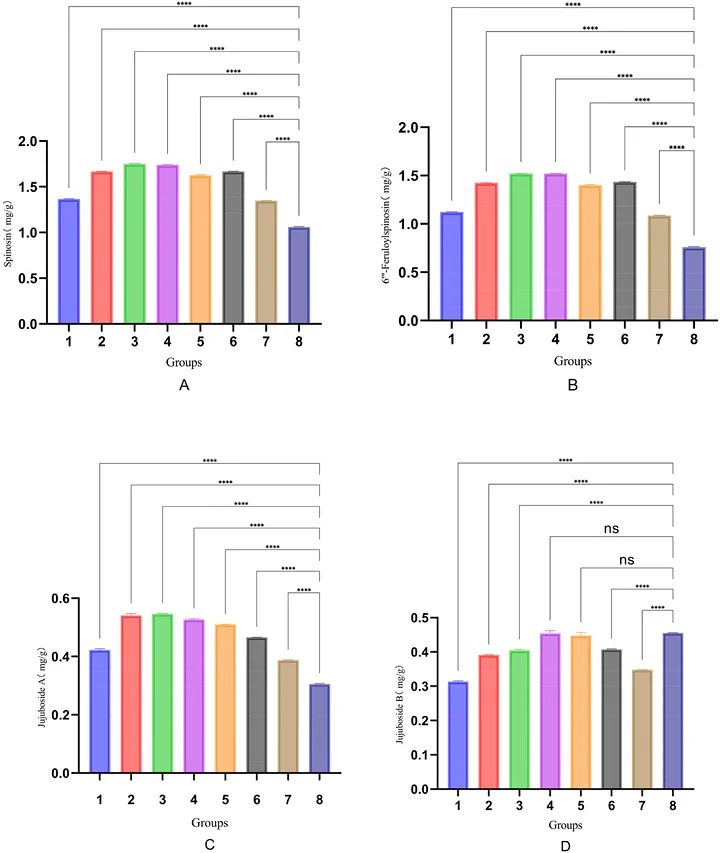
Changes in the contents of spinosin, 6′′′‐feruloylspinosin, jujuboside A, and jujuboside B before and after fermentation of ZSS with different strains of bacteria. 1: *L. acidophilus*; 2: *L. rhamnosus*; 3: *L. plantarum*; 4: *L. paracasei*; 5: *L. acidophilus*: *L. rhamnosus* = 1:1; 6: *L. plantarum*: *L. paracasei* = 1:1; 7: *L. plantarum*: *L. acidophilus*: *L. rhamnosus*: *L. paracasei* = 1:1:1:1 (compared to unfermented sample 8, *p < 0.05, **p < 0.01, and ***p < 0.001).

Changes in spinosin content are shown in Figure 3A. The figure indicates that spinoside levels in fermented ZSS varied significantly across different microbial combinations, with all groups exhibiting a substantial increase compared to the unfermented control. Sample 3 contained 1.7516 mg/g of spinosin, while Sample 8 contained 1.0588 mg/g, representing a 65.43% increase (*p* < 0.05). Changes in 6′′′‐feruloylspinosin content changes are shown in Figure 3B. After fermentation with different bacterial strains, the 6'''‐feruloylspinosin content in ZSS showed a significant increase compared to the unfermented group. Sample No.3 contained 1.5202 mg/g of 6'''‐feruloylspinosin, reaching 100.53% of the original level (*p* < 0.05). Fermented Chinese jujube processed with *Aspergillus purpureus* exhibited increased levels of epicatechin, p‐hydroxybenzoic acid, and chlorogenic acid, showing 16.72‐, 14.05‐, and 6.03‐fold increases compared to the control group [31]. Onion extracts treated with crude enzyme extracts from the soybean paste fungus *Aspergillus kawachii*. showed increased quercetin content. *Aspergillus kawachii*. possesses strong glucosidase activity, capable of hydrolyzing the glycosidic bonds of quercetin glucosides, thereby increasing flavonoid content [32]. In this experiment, various microorganisms hydrolyzed and converted glycosides and compounds structurally similar to spinosin, thereby increasing the content of spinosin and 6′′′‐feruloylspinosin. Moreover, we speculate that the amount of 6′′′‐feruloylspinosin released from plant cells exceeds the amount consumed during its degradation.

Changes in jujuboside A content are shown in Figure 3C. Compared to unfermented ZSS, fermented ZSS showed varying degrees of increase in jujuboside A content. Sample 2 contained 0.5415 mg/g, while Sample 3 contained 0.5463 mg/g, representing a 79.06% increase in Sample 3 (*p* < 0.05). Changes in jujuboside B content are shown in Figure 3D. Sample 8 contained 0.4554 mg/g of jujuboside B. Compared to the unfermented group, fermented samples exhibited reduced jujuboside B content, with Samples 1 and 7 showing significant decreases of 31.11% and 23.56%, respectively. The post‐fermentation jujuboside B content of Samples 4 and 5 showed no significant difference in decrease (*p* > 0.05), while the others decreased significantly compared to unfermented levels (*p* < 0.05). Research indicates that fermenting red ginseng using the *Bifidobacterium animals* subsp. *lactis* LT 19‐2 reduces ginsenosides Rb1, Re, Rc, and Rb3 while increasing Rd, Rh1, F2, and Rg3. This demonstrates that the *β*‐glucosidase produced by microorganisms can break down the sugar portion of these compounds [33]. Microbial activity can lead to either increased or decreased saponins in the fermented product.

### 3.3 Antioxidant Activity Analysis

**FIGURE 4:**
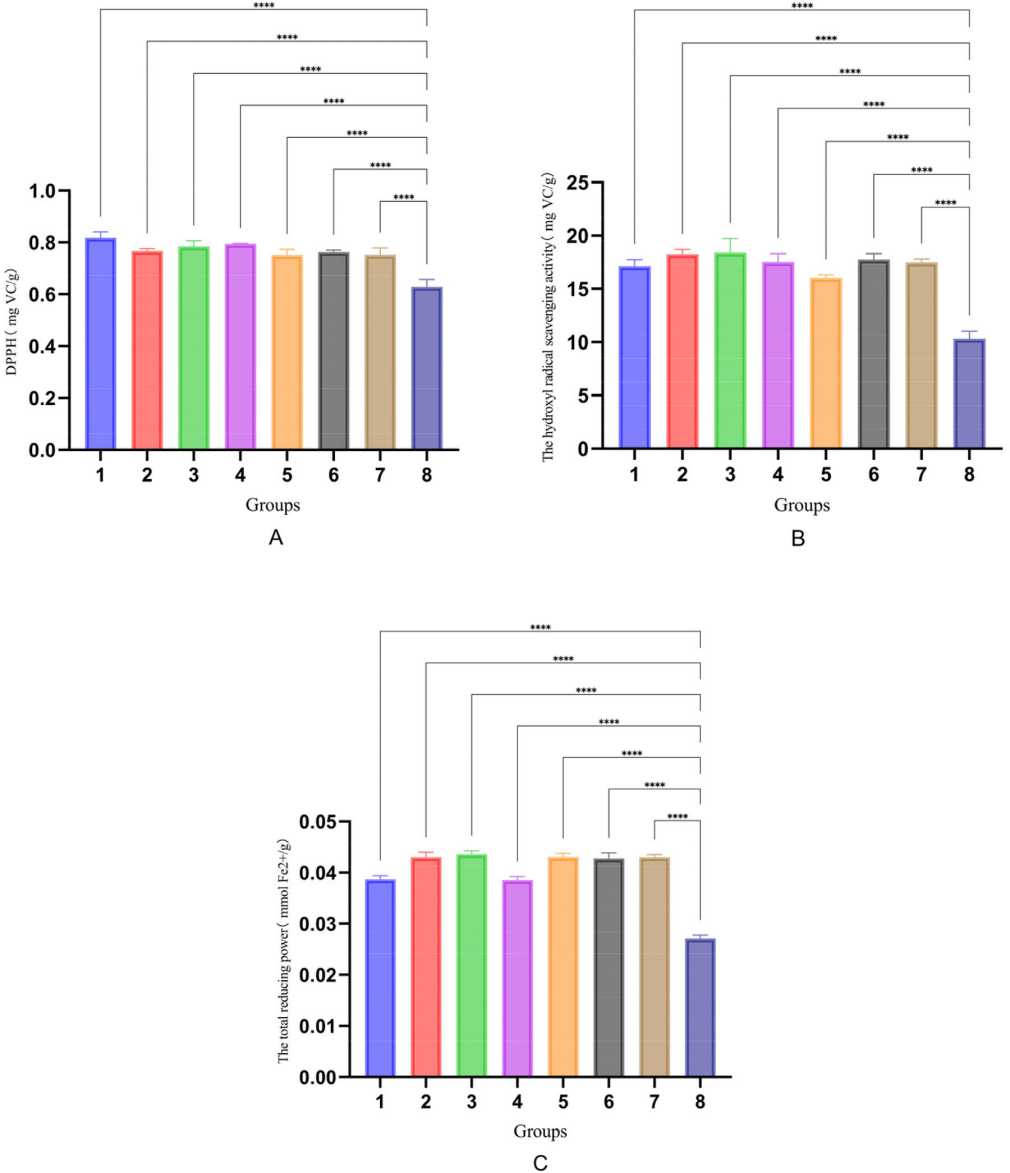
Changes in DPPH radical scavenging activity, hydroxyl radical scavenging activity and total reducing capacity before and after fermentation of ZSS with different strains of bacteria. 1: *L. acidophilus*; 2: *L. rhamnosus*; 3: *L. plantarum*; 4: *L. paracasei*; 5: *L. acidophilus*: *L. rhamnosus* = 1:1; 6: *L. plantarum*: *L. paracasei* = 1:1; 7: *L. plantarum*: *L. acidophilus*: *L. rhamnosus*: *L. paracasei* = 1:1:1:1 (compared to unfermented sample 8, *p < 0.05, **p < 0.01, and ***p < 0.001).

DPPH radical scavenging activity is shown in Figure 4A. The DPPH radical scavenging activity in fermented samples ranged from 0.7515 ± 0.02 to 0.8178 ± 0.02 mg VC/g, all significantly higher than unfermented samples, representing increases of 19.49‐30.04% (*P* < 0.05). Research by Liu et al. demonstrated that fermenting Cistanche deserticola with *L. plantarum* enhanced DPPH radical scavenging capacity to 58.1%, thereby improving fatigue resistance in mice [34]. Research indicates [35] that fermenting bayberry pomace beverages using a mixed fermentation model involving yeast, lactic acid bacteria, and acetic acid bacteria resulted in the detection of gallic acid and protocatechuic acid in all samples, with their concentrations significantly increased. The levels of myricetin and quercetin in the test samples decreased, likely due to damage to their molecular structures caused by substances such as lactic acid and acetic acid produced during fermentation, leading to their decomposition into other compounds. Moreover, both phenolic compounds and flavonoids in the fermented bayberry pomace beverage exhibited DPPH radical scavenging activity, with the antioxidant capacity of the fermented test samples markedly enhanced. As demonstrated in Sections 3.1 and 3.2, TPC and TFC increased in fermented ZSS, consequently boosting their DPPH radical scavenging capacity compared to unfermented samples.

The hydroxyl radical scavenging activity is shown in Figure 4B. The range of 16.5341 ± 0.63∼18.2506 ± 0.36 mg vc/g of Lactobacillus after fermentation was greater than that of the sample before fermentation (*p* < 0.05%).Except for lower values in *L. paracasei*, hydroxyl radical scavenging activity showed minimal variation across different bacterial strains. Pan et al. [36] significantly enhanced the antioxidant capacity of fermented date paste using *Streptococcus thermophilus*. They also analyzed the relationship between antioxidant activity and total phenolic compounds, finding that *Streptococcus thermophilus* fermentation produced more phenolic compounds. The increased phenolic content elevated the antioxidant capacity of the date paste.

The total reducing power is shown in Figure 4C. The total reducing power of Lactobacillus fluctuated between 0.0386 and 0.0436 mmol Fe^2+^/g, representing a 42.44–60.89% increase(*P* < 0.05%) compared to unfermented levels. The iron ion reduction capacity is positively correlated with the hydrogen‐supplying capacity of the reducing agent. During fermentation, reducing agents may form, enhancing the reduction capacity of the fermented samples. Li et al. fermented jujube juice using four probiotics, which improved antioxidant capacity by altering phenolic compounds in the fermented jujube juice [37]. A study on the antioxidant capacity of injera [38] revealed that fermented injera exhibited increased soluble phenolic compounds and soluble flavonoids, with ferulic acid and catechins predominating among the phenolic compounds, significantly enhancing injera's FRAP. These findings are consistent with the increased flavonoid content observed in Sections 3.1 and 3.2 of this experiment. Specifically, 6′′′‐feruloylspinosin can be hydrolyzed by microbial and enzymatic action into spinosin and ferulic acid, leading to elevated levels of both compounds in the samples and thereby enhancing the antioxidant capacity of the test samples.

### 3.4 PcaPca

**FIGURE 5:**
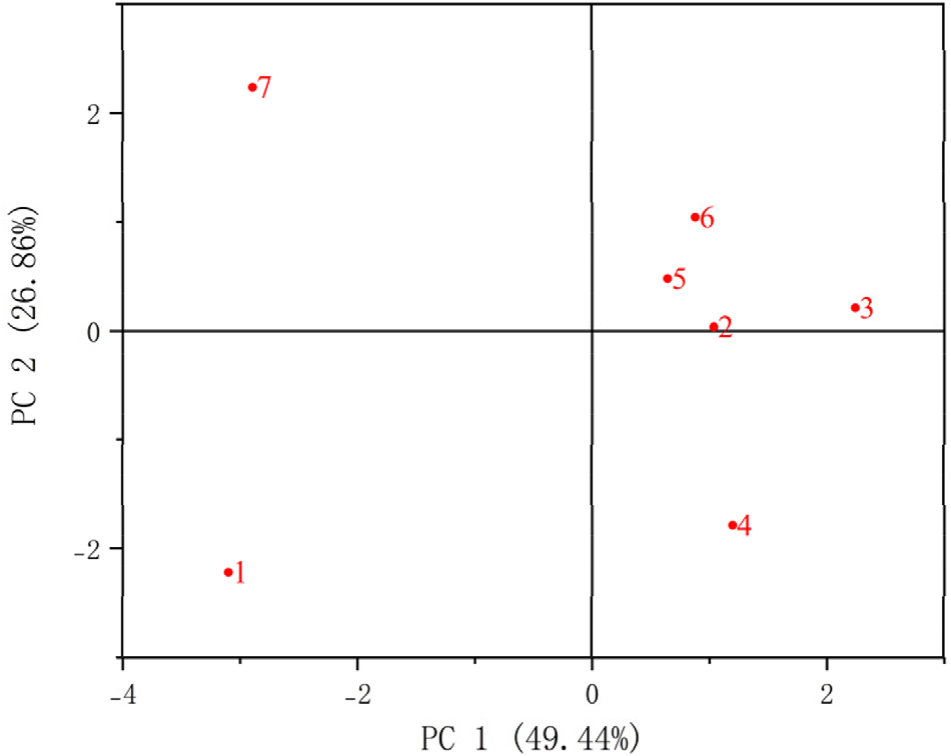
PCA score plot of different *Lactobacillus* strains and their combinations based on nine standardized variables. PC1 and PC2 explained 49.44% and 26.86% of the total variance, respectively. Each point represents one sample. Groups: 1: *L. acidophilus*; 2: *L. rhamnosus*; 3: *L. plantarum*; 4: *L. paracasei*; 5: *L. acidophilus*: *L. rhamnosus* = 1:1; 6: *L. plantarum*: *L. paracasei* = 1:1; 7: *L. plantarum: L. acidophilus*: *L. rhamnosus*: *L. paracasei* = 1:1:1:1.

PCA was performed on nine standardized variables. Three principal components with eigenvalues greater than 1 were extracted, collectively accounting for 91.23% of the total variance (Table 4). PC1 had an eigenvalue of 4.450, explaining 49.44% of the variance, and PC2 had an eigenvalue of 2.418, explaining 26.86% of the variance. The cumulative contribution rate of PC1 and PC2 was 76.30%, and these two components were used to construct the score plot (Figure 4) and loading plot (Figure 5). The PCA score plot (Figure 4) showed a clear separation among the different fermentation groups.

**TABLE 4:** Eigenvalues and variance contribution rates of the extracted principal components.

Principal component	Eigenvalue	Variance (%)	Cumulative variance (%)
PC1	4.4495	49.22%	49.44%
PC2	2.4175	26.86%	76.30%
PC3	1.3436	14.93%	91.23%

**FIGURE 6:**
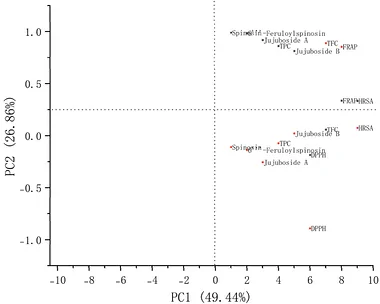
PCA loading plot of nine standardized variables. PC1 and PC2 explained 49.44% and 26.86% of the total variance, respectively. Each point represents one sample. Groups: 1: *L. acidophilus*; 2: *L. rhamnosus*; 3: *L. plantarum*; 4: *L. paracasei*; 5: *L. acidophilus*: *L. rhamnosus* = 1:1; 6: *L. plantarum*: *L. paracasei* = 1:1; 7: *L. plantarum*: *L. acidophilus*: *L. rhamnosus*: *L. paracasei* = 1:1:1:1.

The loading plot (Figure 6) revealed the contributions of individual variables to PC1 and PC2. Spinosin, 6′′′‐feruloylspinosin, jujuboside A, TPC, and jujuboside B showed strong positive loadings on PC1 (all > 0.81), indicating that these five variables were the primary drivers of the separation along PC1. TFC and FRAP exhibited high positive loadings on PC2 (0.888 and 0.853, respectively), whereas DPPH showed a strong negative loading on PC2 (−0.891). The close clustering of spinosin, 6′′′‐feruloylspinosin, jujuboside A, TPC, and jujuboside B in the positive region of PC1 indicated strong positive correlations among these variables. Similarly, TFC and FRAP were closely grouped in the positive region of PC2, suggesting a strong positive correlation between flavonoid content and reducing antioxidant power.

The composite scores, calculated from PC1, PC2, and PC3 weighted by their respective variance contribution rates, are presented in Table 5. *L. plantarum* achieved the highest composite score. Combined with the score plot and loading plot, this result indicates that *L. plantarum* was the optimal fermentation strain because it most effectively enhanced the contents of spinosin, 6′′′‐feruloylspinosin, jujuboside A, and TPC, which were the major positive contributors to PC1—the component explaining the largest proportion of the total variance.

**TABLE 5:** Principal component scores and composite scores.

Sample number	F1	F2	F3	H	Overall ranking
3	2.24	0.21	1.35	1.3654	1
6	0.88	1.05	0.36	0.7708	2
2	1.04	0.04	0.57	0.61	3
5	0.64	0.48	−2.29	0.1035	4
4	1.2	−1.79	−0.57	0.0274	5
7	−2.89	2.24	0.16	−0.8032	6
1	−3.1	−2.22	0.43	−2.0647	7

### 3.5 Pearson Correlation Analysis

**FIGURE 7:**
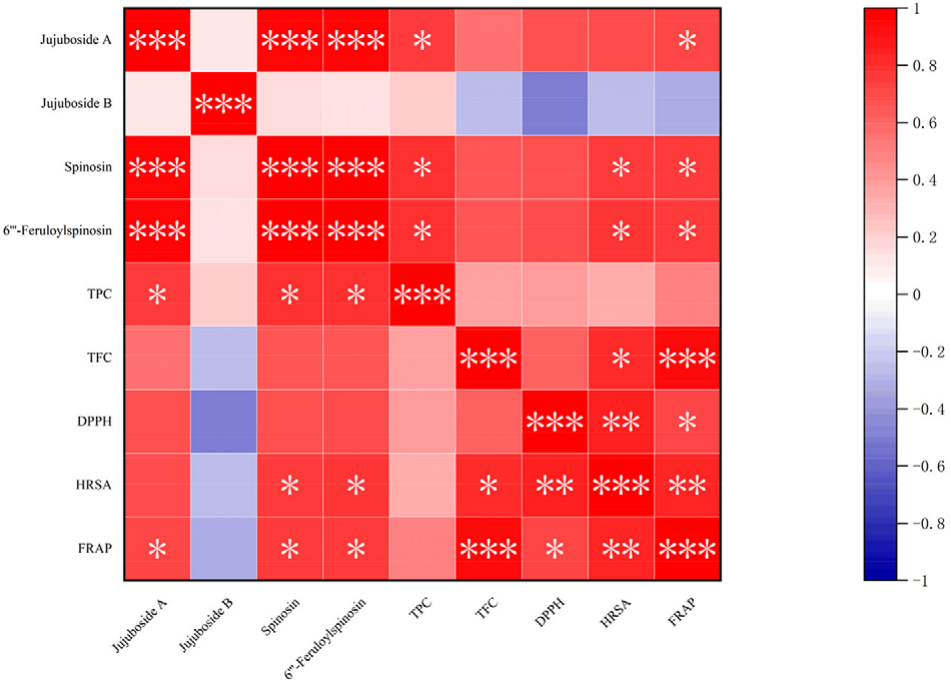
Pearson correlation heatmap of bioactive components and antioxidant activities in fermented ZSS. Red and blue squares indicate positive and negative correlations, respectively. Significance levels: *p < 0.05, **p  <  0.01, ***p < 0.001. Nonsignificant correlations (*p *> 0.05) are not marked.

Pearson correlation analysis was performed to evaluate the relationships among the four quantified compounds (spinosin, 6′′′‐feruloylspinosin, jujuboside A, and jujuboside B), TPC, TFC, and three antioxidant indices (DPPH radical scavenging activity, hydroxyl radical scavenging activity (HRSA), and FRAP). The correlation heatmap is shown in Figure 7.

TFC exhibited significant positive correlations with hydroxyl radical scavenging activity (*p* < 0.05) and FRAP (*p* < 0.05). TPC showed positive but nonsignificant correlations with the three antioxidant indices. Among the four individual compounds, spinosin and 6′′′‐feruloylspinosin displayed significant positive correlations with hydroxyl radical scavenging activity and FRAP (p < 0.05), while jujuboside A showed a significant positive correlation with FRAP (*p* < 0.05). These results indicate that flavonoid compounds are the primary contributors to the antioxidant activity observed in this study.

### 3.6 LC‐MS Results

**FIGURE 8:**
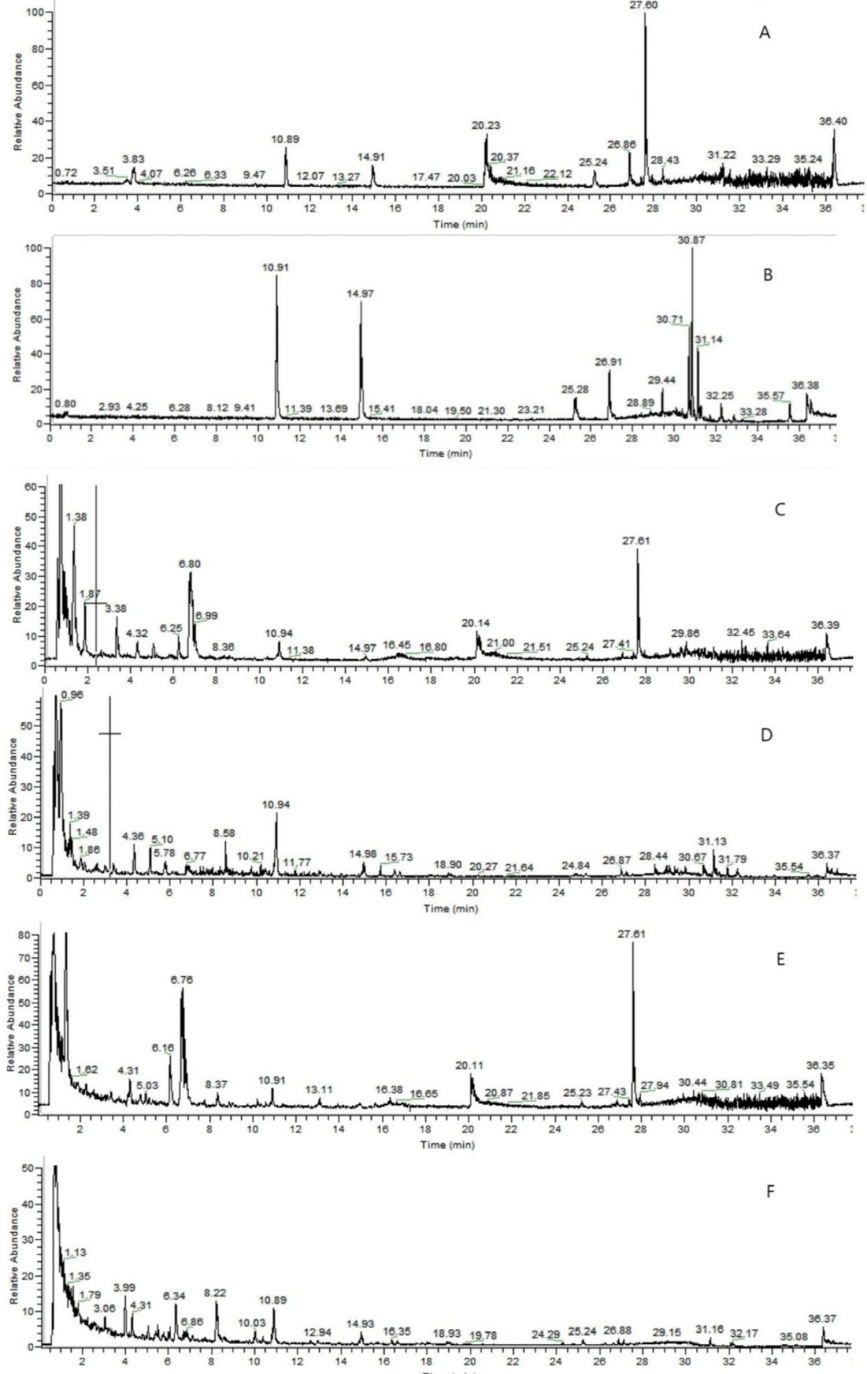
TIC in positive and negative ion mode for mixed control and test products. A, B standards; C, D unfermented; E, F *L. plantarum* fermentation; A, C, E positive ion mode; B, D, F negative ion mode.

Based on Section 3.4, Combination 3 demonstrated the most effective fermentation of ZSS using a single *L. plantarum* strain. Test solutions were prepared using Combination 3 and unfermented ZSS. These test solutions and reference solutions were analyzed under the chromatographic and mass spectrometry conditions specified in Sections 2.4 and 2.5. The resulting total ion chromatograms are shown in Figure 8.

A total of 41 chemical components were identified from the three test solutions. Among these, 41 components were characterized in unfermented ZSS, while 30 chemical components were identified in *L. plantarum*‐fermented ZSS. The main components were alkaloids, flavonoids, saponins, and triterpenoids (Table 6).

**TABLE 6:** UPLC‐Q‐Exactive Orbitrap‐MS /MS mass spectrometry data and identification of chemical constituents in ZSS before fermentation, *L. plantarum* fermented ZSS.

No.	RT Unfermented(min)	RT Fermented(min)	MF	Ion type	Theoretical value *m/z*	Actual value *m/z*	Error/ppm	MS/MS	Chemical composition
1	2.63	—	C_16_H_19_NO_9_	[M+H]^+^	370.1133	370.1129	−1.08	352.1023, 308.1125, 232.0601, 208.0602, 188.0704, 146.0599	3S‐N‐glc‐3‐hydroxy‐indoleacetic acid
2	3	2.98	C_16_H_18_O_9_	[M‐H]^−^	329.0878	329.0879	0.3	167.0338, 152.0102	Pseudolaroside B
3	4.35	4.29	C_23_H_29_NO_8_	[M+H]^+^	448.1966	448.1957	−2.01	286.1429, 269.1162, 237.0895, 209.0958, 175.0751, 107.0493	6‐glc‐coclaurine
4	5.02	4.99	C_16_H_19_NO_8_	[M+H]^+^	354.1183	354.1179	−1.13	54.0805, 188.0703, 174.0546, 146.0598, 128.0493	N‐glc‐indoleacetic acid
5	5.19	5.2	C_19_H_24_NO_3_^+^	M^+^	314.1751	314.1746	−1.59	269.1170, 175.0738, 107.0495	Magnocurarine
6	6.26	6.26	C_17_H_19_NO_3_	[M+H]^+^	286.1438	286.144	0.7	269.1163, 254.0938, 237.0903, 209.0959, 194.0720, 175.0751, 143.0490, 115.9543, 107.0494	Coclaurine
7	6.8	6.81	C_20_H_24_NO_4_+	M^+^	342.1700	342.1697	−0.88	297.1120, 282.0874, 265.0853, 237.0902, 222.1698, 191.0847, 165.0689	Zizyphusine
8	7.66	7.65	C_15_H_14_O_6_	M‐H	289.0717	289.0717	0	221.1810, 203.0705, 187.0385, 109.0279, 97.0278	Catechin/Epicatechin
9	7.72	7.75	C_19_H_24_NO_3_^+^	M+	314.1751	314.1742	−2.86	269.1160, 237.0898, 209.0965, 137.0589, 107.0494	Lotusine
10	10.16	10.17	C_42_H_48_O_23_	[M+H]^+^	921.2659	921.2652	−0.76	726.5487, 479.9072, 351.0861, 327.0860, 151.0388	6'‐(4''‐O‐glc)‐vanilloylspinosin
11	10.34	10.37	C_27_H_30_O_15_	[M+H]^+^	595.1657	595.1654	−0.50	415.0988, 397.0934, 379.0812, 367.0795, 337.0693, 313.0702, 283.0596	Isovitexin‐2‐O‐glucoside
12	10.38	—	C_27_H_30_O_15_	[M‐H]^−^	593.1511	593.1494	−2.87	413.0877, 335.0580,	Vicenin II
13	10:45	10.46	C_42_H_46_O_22_	[M+H]^+^	891.2553	891.2545	−0.90	675.5423, 351.0874, 327.0857, 121.0824	6'''‐(4'''‐O‐glc)‐p‐Hydroxybenzoylspinosin
14	10.72	10.72	C_44_H_50_O_23_	[M+H]^+^	947.2816	947.2801	−1.58	457.8953, 327.0856, 177.0544	6''‐(4'''‐O‐glc)‐feruloylspinosin
15	10.78	10.78	C_32_H_38_O_19_	[M+H]^+^	727.208	727.2077	−0.41	595.1657, 581.1494, 449.1076, 287.0547	Camelliaside B
16	10.86	10.91	C_28_H_32_O_15_	[M‐H]^−^	607.1658	607.1648	1.64	487.1253, 445.1119, 427.1032, 367.0882, 337.0720, 325.0714, 307.0612	Spinosin*
17	10.94	10.94	C_28_H_32_O_15_	[M+H]^+^	609.181	609.181	0	447.1284, 393.2972, 327.0859, 285.0753	Isospinosin
18	11.03	11.01	C_21_H_20_O_10_	[M‐H]^−^	431.0972	431.0981	2.09	413.1302, 395.0926, 341.1089, 311.0988, 269.0877	Isovitexin
19	11.36	11.36	C_22_H_22_O_10_	[M+H]^+^	447.1286	447.1283	−0.67	429.1171, 411.1064, 327.0858, 297.0822, 297.0822	Swertisin
20	11.51	11.50	C_34_H_35_NO_16_	[M+H]^+^	714.2029	714.2026	−0.42	535.5948, 367.1835, 351.0854, 327.0858, 297.0734	6'''‐Pyridyloylspinosin
21	12.26	12.25	C_41_H_46_O_20_	[M+H]^+^	859.2655	859.2649	−0.70	379.4897, 327.0833, 297.0746, 251.0902, 219.0641	6''‐(2''',3''',4''',5'''‐tetramethoyl)‐(E)‐cinnamoylspinosin
22	12.42	12.42	C_36_H_38_O_18_	[M+H]^+^	759.2131	759.2125	−0.79	639.1946, 393.2791, 327.1219, 151.0351	6'''‐Vanilloylspinosin
23	12.88	12.89	C_35_H_36_O_17_	[M+H]^+^	729.2025	729.202	−0.69	609.1881, 447.3463, 327.0851, 285.0747	6'''‐p‐Hydroxybenzoylspinosin
24	13.14	—	C_18_H_19_NO_2_	[M+H]^+^	282.1485	282.1489	1.42	265.1220, 235.1690, 207.0650, 191.0710, 165.0704	N‐Methylasimilobine
25	13.4	13.37	C_37_H_38_O_18_	[M‐H]^−^	769.1974	769.1988	1.82	593.1559, 413.1306, 353.1093, 293.0882	isovitexin‐2″‐O‐(6‐feruloyl)‐glucopyranoside
26	14.24	—	C_27_H_30_O_15_	[M‐H]^−^	593.1501	593.1505	0.67	556.3484, 413.0867, 357.0375, 311.0555, 293.0455, 89.0230	isovitexin‐2″‐O‐glucopyranoside
27	14.37	14.38	C_39_H_42_O_19_	[M+H]^+^	815.2393	815.2388	−0.61	695.7874, 609.1854, 591.2039, 447.3468, 393.2973, 297.1672, 207.0651	6'''‐Sinapoylspinosin
28	14.86	—	C_37_H_38_O_17_	[M+H]^+^	755.2182	755.2177	−0.66	635.1747, 489.1383, 411.1069, 327.0864	6'''‐p‐Coumaloylspinosin
29	14.98	14.95	C_38_H_40_O_18_	[M+H]^+^	785.2287	785.2281	−0.76	665.1860, 447.1288, 429.1179, 393.2970, 351.0861, 327.0862, 297.0762, 177.0544	6'''‐Feruloylspinosin*
30	15.67	15.65	C_43_H_52_O_19_	[M+H]^+^	873.3176	873.3162	−1.60	608.1909, 330.8001, 327.0848, 297.0756, 247.1313	6'''‐Dihydrophaseoyl spinosin
31	16.57	16.31	C_44_H_49_NO_22_	[M+H]^+^	944.2819	944.2809	−1.06	764.2186, 602.1642, 429.1154, 411.1079, 393.0961, 351.0858, 327.0857, 297.0751, 146.0598	6'''‐O‐(3‐glc‐indole‐acetyl)spinosin
32	17.18	17.22	C_43_H_50_O_19_	[M+H]^+^	871.3019	871.3011	−0.92	792.2901, 351.0858, 327.0844, 297.0754	6'''Phaseoylspinosin
33	19.45	—	C_64_H_106_O_32_	[M‐H]^−^	1385.6583	1385.6558	−1.8	1367.2810, 1081.5203, 949.4087	Protojujuboside A
34	20.23	—	C_58_H_96_O_27_	[M‐H]^−^	1223.6055	1223.6071	1.31	1205.3921, 1090.5417, 919.5618, 787.2104, 479.1752	Protojujuboside B
35	21.18	—	C_58_H_96_O_27_	[M‐H]^−^	1223.6066	1223.6066	0	1189.7172, 920.4317, 787.4090, 625.3634	Protojujuboside B_1_
36	25.24	25.24	C_58_H_94_O_26_	[M‐H]^−^	1207.6106	1207.6119	1.08	1073.5547, 1055.4466, 911.4515, 749.2771, 603.3939, 455.3516	Jujuboside A*
37	26.87	26.89	C_52_H_84_O_21_	[M+H]^+^	1045.5583	1045.5565	−1.72	895.5035, 881.4883, 749.4433, 733.4515, 697.4304,601.4094, 587.3939, 533.3621, 473.3625, 455.3515, 437.3412, 369.2785	Jujuboside B*
38	27.11	27.12	C_52_H_84_O_21_	[M+H]^+^	1045.5578	1045.5565	−1.24	1027.5466, 913.5175, 899.4969, 751.4620, 733.4523, 697.4310, 587.3940, 473.3625, 455.3517, 437.3412	Jujuboside III
39	29.98	—	C_30_H_46_O_5_	[M‐H]^−^	485.3262	485.327	1.65	423.3264	Ceanothic acid
40	30.81	—	C_30_H_48_O_3_	[M‐H]^−^	455.3531	455.3532	0.22	423.2916	Betulinic acid*
41	34	—	C_30_H_46_O_3_	[M‐H]^−^	453.3374	453.3390	3.53	428.0555, 291.0825	Betulonic acid

*Note*: — indicates not detected; * indicates standard sample.

#### 3.6.1 Flavonoids

22 flavonoids and flavonol glycosides were identified in fermented ZSS (8, 10, 12, 13, 14, 15, 16, 17, 18, 19, 20, 21, 22, 23, 25, 26, 27, 28, 29,30, 31, 32). From *L. plantarum*‐fermented ZSS, 19 compounds were identified (8, 10, 13, 14, 15, 16, 17, 18, 19, 20, 21, 22, 23, 25, 27, 29, 30, 31, 32). These compounds primarily exist as molecular ions in the [M+H]^+^ and [M‐H]^−^ forms. Most flavonoids in ZSS are flavone glycosides, which readily shed various substituents during fragmentation to form the flavone aglycone. In secondary spectra, fragment ion formation is predominantly associated with the loss of sugar residues, H_2_O, ‐CH_3_, ─CO, and RDA cleavage. Fragmentation typically occurs on the hexose sugar directly linked to the aglycone, yielding fragments with mass units of 60(─CHO─CH_2_OH), 90(─CHOH─CHO─CH_2_OH),120(─CHOH─CHOH─CHO─CH_2_OH) mass units. The following analysis and identification are performed using compound 16 as an example.

**FIGURE 9:**
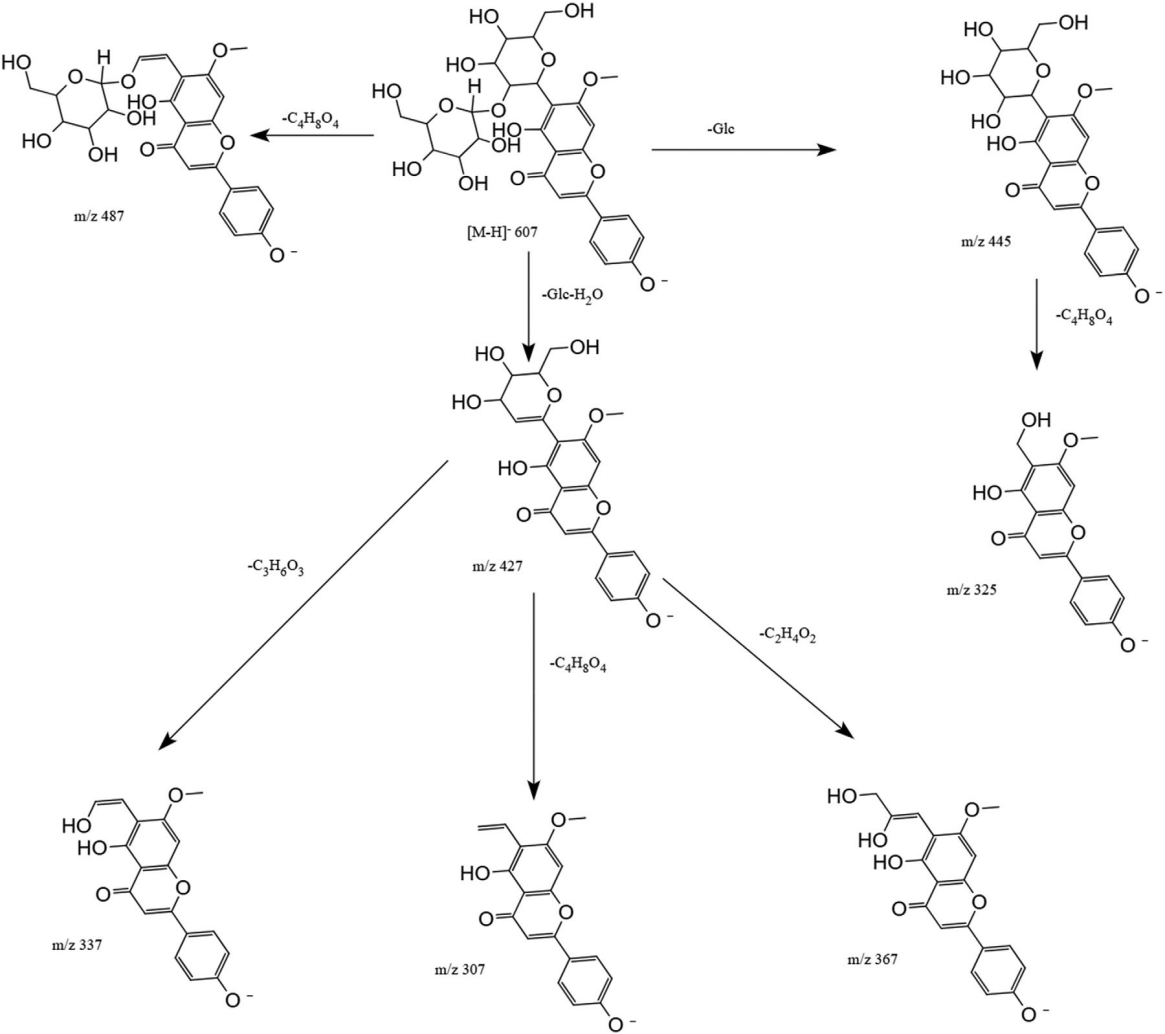
Possible Cleavage Pathways of spinosin.

In positive ion detection mode, the retention times t_R_ for ZSS before fermentation and *L. plantarum*‐fermented ZSS were 10.86 min and 10.91 min, respectively. The m/z of the [M+H]^+^ peak had a m/z of 609.1814, suggesting a molecular formula of C_28_H_32_O_15_. In the [M+H]^+^ secondary mass spectrum, prominent fragment ions included m/z 489.1387, 447.1282, 429.1177, 369.0690, 339.2141, and 309.0853. Using m/z 609.1814 as the parent ion for secondary mass spectrometry analysis, *m/z* 489 in the secondary mass spectrum of compound 12 is inferred to originate from [M+H‐C_4_H_8_O_4_]^−^; the loss of one glucose molecule yields the fragment ion [M+H‐Glc]^+^ at *m/z* 447, and the loss of one C_4_H_8_O_4_ molecule from [M+H‐Glc]^+^ forms the fragment ion [M+H‐Glc‐C_4_H_8_O_4_]^+^ fragment ion, producing a base peak at this point; [M+H‐Glc]^+^ loses one molecule of H_2_O to form the [M+H‐Glc‐H2O]^+^fragment ion at *m/z* 429, then loses C_2_H_4_O_2_ to form 369; [M+H‐Glc‐H_2_O]^+^ loses 1 molecule of C_3_H_6_O_3_ to form [M+H‐Glc‐H_2_O‐C_3_H_6_O_3_] at *m/z* 339^+^ Fragment ion; [M+H‐Glc‐H_2_O]^+^ loses one molecule of C_4_H_8_O_4_ to form [M+H‐Glc‐H_2_O‐C_4_H_8_O_4_]^−^ at *m/z* 309^+^ Fragment ion; analysis of its cleavage process reveals regular fragmentation peaks decreasing by 60, 90, and 120 mass units. Based on the above information and references [39], this compound is likely spinosin(Figure 9).

#### 3.6.2 Saponins and Triterpenoids

9 saponins and triterpenoids (33, 34, 35, 36, 37, 38, 39, 40, 41) were identified in unfermented ZSS 3 saponins and triterpenoids (36,37,38) were identified in *L. plantarum*‐fermented ZSS. ZSS saponins primarily comprise tetraterpenoid saponins and pentaterpenoid saponins, with dammarane‐type tetraterpenoids constituting the majority. Secondary fragment ions of saponins and terpenoids are typically associated with the loss of a series of sugar moieties. Compound 36 was selected as an example for identification analysis.

**FIGURE 10:**
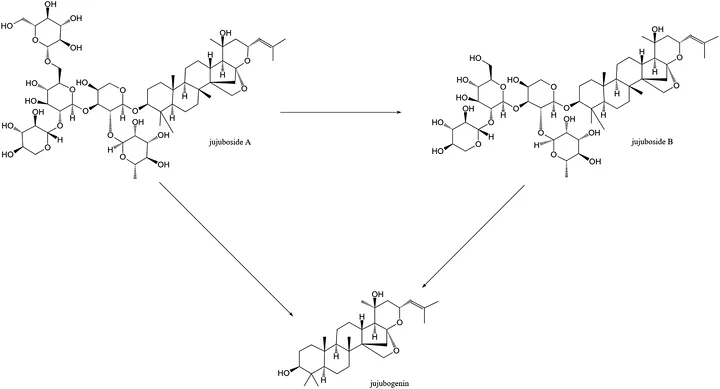
Possible Hydrolysis Pathways of jujuboside A.

In positive ion mode, the retention times before fermentation and after *L. plantarum* fermentation were 25.24 min, 25.24 min, and 25.23 min, respectively. The m/z values of the precursor ions [M+H]^+^ and [M‐H]^−^ were 1207.6106 and 1205.5963, respectively, suggesting a molecular formula of C_58_H_94_O_26_. Secondary mass spectrometry analysis was performed using [M‐H]^−^ as the parent ion. The resulting secondary mass spectrum shows distinct fragment ions:1073[M‐H‐Xyl]^−^ represents the parent ion losing one xylose molecule, followed by sequential sugar losses. 911 indicates loss of one glucose, then another glucose to form 749, 603 results from losing one rhamnose, followed by arabinose loss to form 455. Finally, 1055 originates from [M‐H‐H_2_O‐Xyl]^−^ is a fragment ion formed by the parent ion losing one molecule of water followed by one molecule of xylose. Based on literature [40], this compound is tentatively identified as jujuboside A (Figure 10).

## 4 Speculation on the Mechanism of *L. plantarum* Fermentation of ZSS

*L. plantarum* possesses a remarkably diverse enzymatic repertoire, with its genome encoding 133 carbohydrate‐active enzymes (CAZymes) spanning glycoside hydrolases (GH) [41], glycosyltransferases, carbohydrate esterases (CE) [42], and auxiliary activity enzymes [43]. Key functional enzymes include GH13 α‐amylases, GH1 β‐glucosidases, GH78 α‐rhamnosidases, and CE1 feruloyl esterases, collectively providing the molecular basis for simultaneous degradation of multiple substrate types during fermentation of ZSS.

This diverse enzymatic machinery facilitates the release of bioactive compounds from the cell wall matrix. During fermentation, β‐glucosidases and feruloyl esterases hydrolyze polysaccharide‐phenolic acid complexes and ester bonds linking phenolic acids to cell wall hemicellulose, releasing bound constituents into the free state. Studies have demonstrated that enhanced β‐glucosidase activity in *L. plantarum* resulted in a 19.05% increase in total phenolics, a 54.17% increase in total flavonoids, and an approximately threefold increase in free ferulic acid in fermented jujube juice [44].

Furthermore, the glycoside hydrolase system directly transforms flavonoid glycosides, saponins, and phenolic compounds in ZSS into more bioactive forms. β‐Glucosidases cleave β‐glucosidic bonds in flavonoid glucosides to yield aglycones [45], while α‐rhamnosidases selectively hydrolyze rhamnosyl moieties with strict specificity toward α‐1,6‐glycosidic linkages [43]. For jujubosides, sequential deglycosylation generates secondary saponins with improved bioavailability. In the present study, the contents of spinosin, jujuboside A, and other components also increased after fermentation with *L. plantarum*.

The superior performance of *L. plantarum* compared with the other three tested strains can be attributed to several strain‐specific advantages. First, *L. plantarum* possesses a larger genome and a more diverse repertoire of carbohydrate‐active enzymes than *L. acidophilus*, *L. rhamnosus*, and *L. paracasei*, enabling it to simultaneously hydrolyze multiple types of glycosidic and ester bonds in ZSS [46, 47, 48]. Second, *L. plantarum* exhibits higher tolerance to phenolic compounds, which are abundant in ZSS and can inhibit the growth and metabolic activity of other *Lactobacillus* species. This allows *L. plantarum* to maintain robust enzyme production throughout the fermentation process [49, 50, 51]. Third, *L. plantarum* produces higher levels of organic acids, particularly lactic acid, which synergistically act with enzymes to disrupt the plant cell wall matrix and facilitate the release of bioactive compounds [50, 52, 53]. These combined advantages—enzymatic diversity, phenolic tolerance, and acid production—collectively explain why *L. plantarum* achieved the highest increases in spinosin, 6′′′‐feruloylspinosin, jujuboside A, TPC, TFC, and antioxidant activity among the four strains tested.

The active components of TCM are primarily distributed in the cytoplasm of plant roots, stems, and seed cells. Plant cell walls, mainly composed of cellulose, hemicellulose, and lignin, inhibit the release of natural products, reducing their absorption and utilization rates. Probiotics can produce hydrolases, particularly cellulase and ligninase, which degrade cell walls to promote the release of natural products from TCM [54]. These natural products include flavonoids, glycosides, terpenoids, and organic acids. Compounds in ZSS are predominantly flavonoids and saponins. It is hypothesized that during fermentation, enzymes produced by *L. plantarum* degrade the cell walls of ZSS, enhancing the solubility of active substances [19].

**FIGURE 11:**
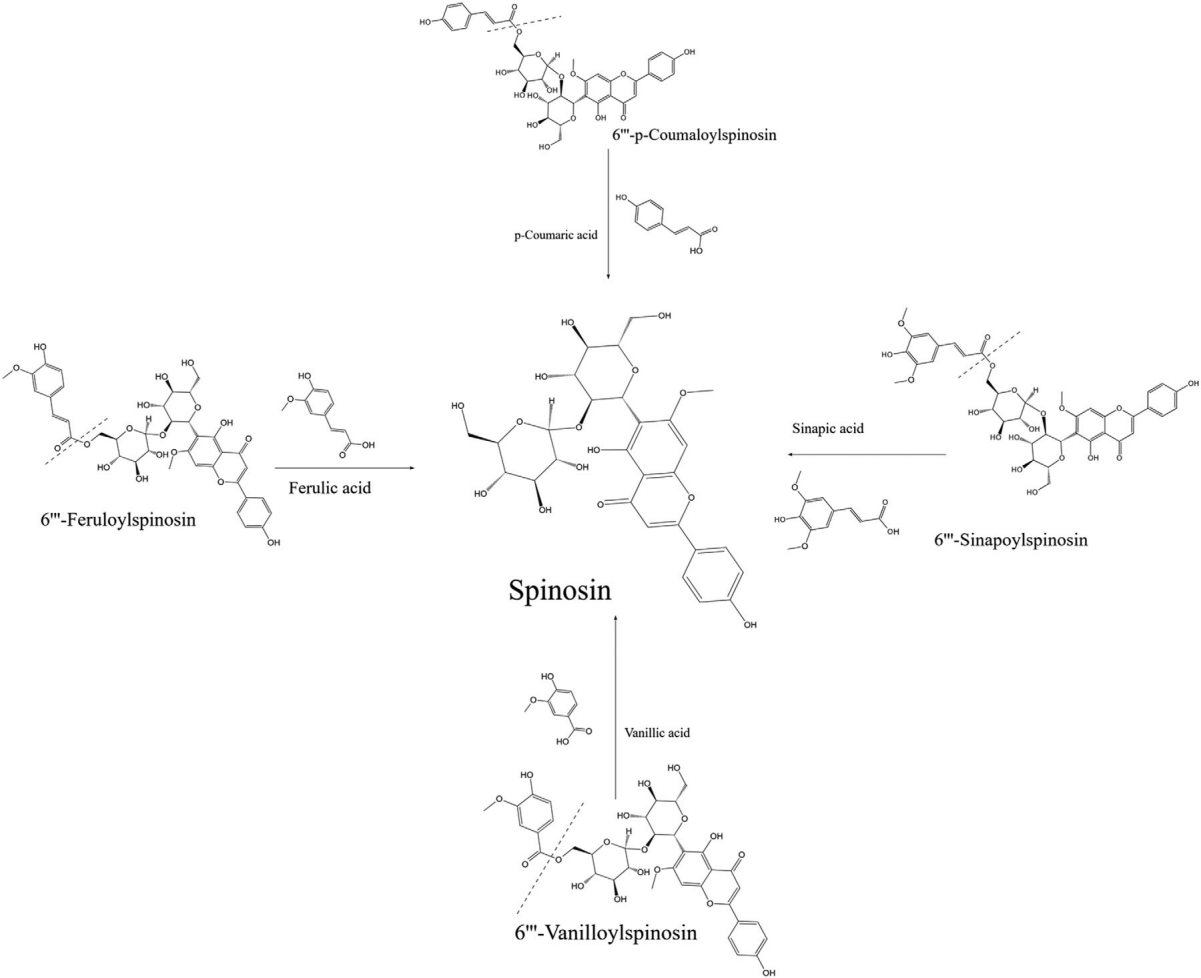
Possible degradation pathways of 6′′′‐Vanilloylspinosin, 6′′′‐Sinapoylspinosin, 6′′′‐p‐Coumaloylspinosin and 6'''‐Feruloylspinosin.

Enzymes produced by *L. plantarum* can catalyze the hydrolysis of various C─O bonds, N─C bonds, acyl groups, and glucosyl groups [55], leading to changes in the types and contents of natural products. Spinosin undergoes hydrolysis by enzymes to release one glucose molecule, yielding Swertisin (19). Swertisin exhibits antioxidant capacity and enhances nematode survival under oxidative stress [56]. Natural products possess antioxidant properties, and the increased content of these compounds in fermented ZSS enhances their antioxidant capacity. Fermented ZSS exhibit elevated spinosin content, potentially due to 6′′′‐Vanilloylspinosin (22), 6′′′‐Sinapoylspinosin (27), 6′′′‐p‐Coumaloylspinosin (28) and 6'''‐Feruloylspinosin (29). These acyl groups are hydrolyzed by ferulic acid esterase produced by *L. plantarum*, yielding spinosyn [57] (Figure 11). Additionally, *Bifidobacterium animalis* subsp. *lactis* LT 19‐2 (LT 19‐2) converts the major ginsenosides Rb2 and Rb3 in red ginseng extract into rare ginsenosides Rd, Rh1, F2, and Rg3 [33]. Microorganisms can produce glycoside hydrolases such as α‐glucosidase, α‐galactosidase, and α‐*L*‐rhamnosidase [29]. These enzymes hydrolyze jujuboside A (36), which consists of a saponin aglycone and five sugar residues (arabinose, rhamnose, xylose, and two glucose units). It readily undergoes enzymatic hydrolysis to release one glucose molecule, yielding jujuboside B (37). The glycosidic bond of jujuboside B can be further hydrolyzed, leading to a reduction in saponin content [58]. Saponins exhibit low bioavailability in the gut. Hydrolytic and reductive reactions by gut microbiota increase the lipophilicity of glycosides, enhancing intestinal absorption of saponins [59]. Phenolic compounds and flavonoids contain one or more aromatic rings bearing hydroxyl groups, which can donate hydrogen atoms to free radicals, thereby terminating radical chain reactions. The enzymatic hydrolysis of flavonoid glycosides and saponins during fermentation releases aglycones, which possess more free hydroxyl groups than their glycosylated precursors. This increase in free phenolic hydroxyl groups enhances the hydrogen‐donating ability of the fermented products, as reflected by the strong positive loadings of TPC and TFC on PC2 and their significant positive correlations with FRAP and DPPH in the PCA loading plot and correlation heatmap.

The reduction in the total number of detected metabolites after fermentation is likely due to the enzymatic hydrolysis of large glycosides and saponins into smaller aglycones and phenolic compounds, some of which may fall below the detection threshold or co‐elute with other peaks. Importantly, this decrease in metabolite number was accompanied by increases in the contents of key bioactive compounds (spinosin, 6′′′‐feruloylspinosin, and jujuboside A) and enhanced antioxidant capacity, indicating that fermentation directed the chemical transformation toward more bioactive forms rather than simply causing a loss of components.

In summary, the enzymatic hydrolysis of flavonoid glycosides and saponins by *L. plantarum* not only alters the chemical composition of ZSS but also converts these compounds into forms with enhanced antioxidant activity, thereby establishing a direct relationship between metabolite transformation and the improved functional properties of fermented ZSS.

## 5 Conclusion

*L. plantarum* was identified as the optimal strain for fermenting ZSS through PCA‐based multi‐indicator screening. *L. plantarum* fermentation increased the contents of spinosin, 6′′′‐feruloylspinosin, and jujuboside A. TFC exhibited significant positive correlations with hydroxyl radical scavenging activity and ABTS radical scavenging activity, while TPC showed positive but nonsignificant correlations with the three antioxidant indices. UPLC‐Q‐Exactive Orbitrap‐MS profiling combined with the observed compositional changes suggested that glycosidases and feruloyl esterase secreted by *L. plantarum* hydrolyze glycosidic, acyl, and ester bonds, converting flavonoid glycosides and saponins into aglycones and free phenolics, thereby enhancing the antioxidant capacity of fermented ZSS. This integrated strategy combining PCA, targeted quantification, untargeted chemical profiling, and antioxidant evaluation provides a methodological reference for strain selection in fermented traditional Chinese medicine.

## Author Contributions

**Jiafu Wan**: writing – original draft, data curation, methodology. **Yunda Wang**: writing – original draft, investigation, resources. **Xiaoxue Yang**: formal analysis, supervision, software. **Qingmei Guo**: writing – review and editing, project administration, funding acquisition.

## Funding

This research was supported by the scientific research fund of Shandong University of Traditional Chinese Medicine (No. KYZK2024Z03).

## Conflicts of Interest

The authors declare no conflicts of interest.

## Supporting information




**Supporting File 1**: cbdv71686‐sup‐0001‐SuppMat.docx

## Data Availability

The data that support the findings of this study are available from the corresponding author upon reasonable request.
