# Supplementary material for: Qualitative Analysis of Chemical Composition Changes Before and After Lactobacillus Fermented Ziziphi Spinosae Semen by LC‐MS and Prediction of Fermentation Mechanism
Source: Chem Biodivers. 2026 Sep 23;23(9):e71686. doi: 10.1002/cbdv.71686 (PMC13599462; doi:10.1002/cbdv.71686)
Supplement: Supplementary file 1 [file CBDV-23-e71686-s001.docx]

Figure S1. Representative chromatograms of (a) spinosin, (b) 6′′′-feruloylspinosin, (c) jujuboside A, (d) jujuboside B. Panels (A) and (C) were detected by DAD at 335 nm; panels (B) and (D) were detected by ELSD.
